# Theoretical Investigation of the Lattice Thermal Conductivities of II–IV–V_2_ Pnictide Semiconductors

**DOI:** 10.1021/acsaelm.3c01242

**Published:** 2023-11-22

**Authors:** Victor Posligua, Jose J. Plata, Antonio M. Márquez, Javier Fdez. Sanz, Ricardo Grau-Crespo

**Affiliations:** † Departamento de Química Física, Facultad de Química, Universidad de Sevilla, Seville 41012, Spain; ‡ Department of Chemistry, University of Reading, Whiteknights, Reading RG6 6DX, U.K.

**Keywords:** thermal conductivity, thermoelectric, Boltzmann transport equation, density functional theory, pnictides, chalcopyrites, CdGeAs_2_

## Abstract

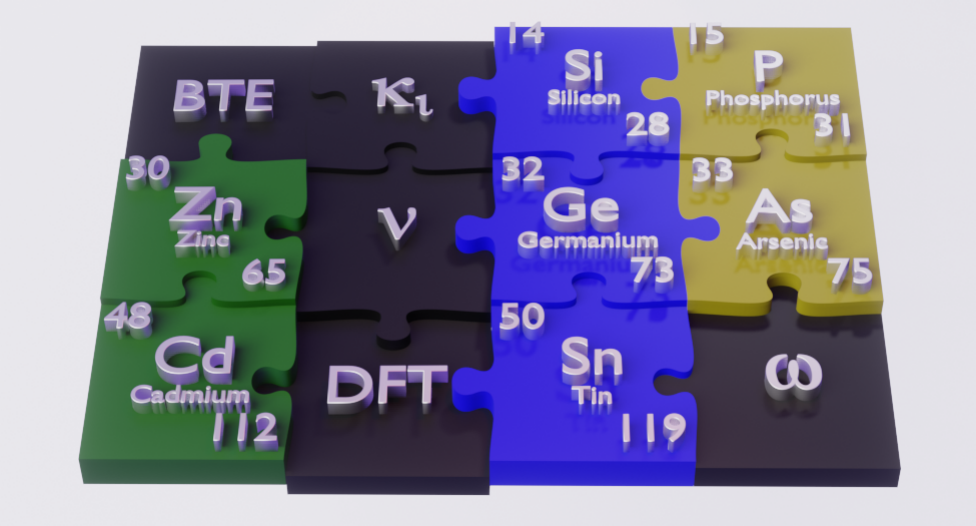

Ternary pnictide semiconductors with II–IV–V_2_ stoichiometry hold potential as cost-effective thermoelectric materials with suitable electronic transport properties, but their lattice thermal conductivities (κ) are typically too high. Insights into their vibrational properties are therefore crucial to finding strategies to reduce κ and achieve improved thermoelectric performance. We present a theoretical exploration of the lattice thermal conductivities for a set of pnictide semiconductors with ABX_2_ composition (A = Zn, Cd; B = Si, Ge, Sn; and X = P, As) using machine-learning-based regression algorithms to extract force constants from a reduced number of density functional theory simulations and then solving the Boltzmann transport equation for phonons. Our results align well with available experimental data, decreasing the mean absolute error by ∼3 W m^–1^ K^–1^ with respect to the best previous set of theoretical predictions. Zn-based ternary pnictides have, on average, more than double the thermal conductivity of the Cd-based compounds. Anisotropic behavior increases with the mass difference between A and B cations, but while the nature of the anion does not affect the structural anisotropy, the thermal conductivity anisotropy is typically higher for arsenides than for phosphides. We identify compounds such as CdGeAs_2_, for which nanostructuring to an affordable range of particle sizes could lead to κ values low enough for thermoelectric applications.

## 1 Introduction

There are two major groups of ternary ABX_2_ semiconductors with chalcopyrite structure: ternary chalcogenides with I–III–VI_2_ composition (A = Cu, Ag; B = Fe, Al, Ga, In, Tl; and X = S, Se, Te) and ternary pnictides with II–IV–V_2_ composition (A = Zn, Cd; B = Si, Ge, Sn; and X = P, As, Sb). Pnictide chalcopyrites, like their chalcogenide counterparts, constitute a remarkably versatile family of semiconducting materials. These materials can be synthesized with cost-effectiveness, and their composition can be extensively adjusted due to their ability to accommodate different cations and anions.

The II–IV–V_2_ ternary pnictides have been studied theoretically and experimentally for their potential applications in optoelectronic devices, including solar cells, due to their stability, dopability, high carrier mobility, and favorable optical absorption/emission properties.^1−5^ Some of these properties also make them interesting materials for thermoelectric applications. Pnictide compounds often exhibit favorable electronic structures that enhance electrical transport, such as the coexistence of flat and highly dispersive bands near the Fermi level, which has motivated previous investigations of their thermoelectric properties.^6−12^

In addition to the high mobility, dopability, and favorable electronic properties, achieving high thermoelectric performance requires low thermal conductivity (κ). Due to the semiconducting nature of the materials, the electronic contribution to κ is small, and the lattice contribution is dominant (hereafter, we refer to the lattice thermal conductivity as κ, ignoring the electronic contribution). Unfortunately, ternary pnictide semiconductors with a chalcopyrite structure tend to have high κ values in bulk form. For example, ZnBX_2_ compounds, with B = Si, Ge, or Sn, and X = P or As, have been predicted to exhibit excellent electron transport properties.^9^ However, measured bulk thermal conductivities are very high, e.g., up to ∼35 W m^–1^ K^–1^ for single-crystal ZnGeP_2_ at room temperature.^13^ Still, variations of these compositions (e.g., replacing Zn with Cd) and nanostructuring effects may lead to much lower values of κ, particularly at higher temperatures. Therefore, gaining a systematic understanding of phonon structure and transport in these pnictide semiconductors, as a function of composition and temperatures, and exploring the impact of nanostructuring on thermal conductivity become crucial for improving the potential of these materials for thermoelectric (as well as other) applications.

Given the sensitivity of κ to the material’s synthetic procedure, which influences grain size and defect chemistry, caution is needed when thermal conductivity trends are interpreted between experimental measurements at different compositions conducted under dissimilar conditions. Computer modeling facilitates a direct comparison of intrinsic thermal conductivity behavior across various compositions and temperatures. Unfortunately, accurately predicting the lattice thermal conductivity poses computational challenges. One of the most accurate approaches relies on solving the Boltzmann’s transport equation (BTE) for phonons,^14^ which requires calculating second- and third-order interatomic force constants (IFCs). Conventionally, these IFCs are determined by computing atomic forces in supercells for each symmetrically distinct displacement of atomic positions using density functional theory (DFT).^1,6,15−19^ However, obtaining third-order IFCs in this way requires a considerable number of DFT calculations, making this step a bottleneck in the first-principle prediction of κ.^6,15,16,18−21^ Recently, innovative algorithms have emerged that expedite the calculation of IFCs by leveraging machine learning and related techniques to enable the extraction of IFCs from a much smaller set of DFT calculations.^22−25^ These advancements pave the way for the accurate calculation of κ across a wide range of compositions, as we have shown before for chalcopyrite chalcogenides^26−28^ and will demonstrate here for the chalcopyrite pnictides.

In this work, we have theoretically investigated the thermal conductivity of a range of pnictide semiconductors with composition ABX_2_, where A = Zn, Cd, Si, Ge, and X = P or As. Additionally, two compositions with B = Sn (CdSnAs_2_ and CdSnP_2_) are included, as they are also known experimentally. We have excluded compositions for which a large degree of cation disorder is expected (e.g., ZnSnAs_2_ and ZnSnP_2_) as they would require a theoretical framework beyond our current approach to deal with the effect of alloy scattering of phonons. We will compare with experimental values whenever available^13,29−37^ to demonstrate the accuracy of our calculations and then make systematic predictions for a comprehensive range of compositions, temperatures, and particle sizes.

## 2 Methodology

### 2.1 DFT-Based Geometry Optimization and Force Evaluations

DFT calculations were conducted using the Vienna Ab Initio Simulation Package (VASP) code,^38,39^ which uses a planewave expansion of the valence wave functions, together with the projector-augmented wave (PAW) method to account for core–valence interactions.^39^ The number of valence electrons for each atom was determined based on the standards suggested by Calderon et al.^40^ Energies and forces were computed using the Perdew, Wang, Ernzerhof (PBE) generalized gradient approximation (GGA) functional,^41^ adding Grimme’s D3 van der Waals corrections.^42^ The kinetic energy cutoff of the plane-wave basis set expansion was set at 500 eV, which is 25% above the standard value for the chosen PAW potentials to reduce Pulay stress errors. Equilibrium structures were found by energy minimization until the forces on all atoms were less than 10^–7^ eV Å^–1^ (the strict criterion for force convergence was needed for accurate phonon calculations). To minimize noise in the forces, an additional support grid was used for the evaluation of augmentation charges. Geometry optimizations were initially performed on the tetragonal conventional cells consisting of 16 atoms. The forces required to calculate the IFCs were then obtained using a 4 × 4 × 2 supercell (512 atoms), and in this case, reciprocal space integrations were performed solely at the Γ point. We checked that increasing the grid density to a Γ-centered 2 × 2 × 2 mesh did not notably impact the results.

### 2.2 Force Constant Prediction and Machine Learning Regression

The hiPhive package,^23^ based on machine-learning regression algorithms, enabled the extraction of second-, third-, and fourth-order force constants within optimized cutoff distances, from the DFT-calculated forces. Fourth-order force constants do not have a direct effect on the BTE model used here (see the discussion below); however, their inclusion slightly improved the regression for the force constant potential (FCP) model. Multilinear regression to the DFT forces was carried out to obtain the force constants using the recursive feature elimination (RFE) algorithm, which efficiently selects the most relevant features for the regression.^43^ The convergence of the FCP model parameters (number of distorted structures and cutoff distances) was tested by evaluating the variation in the lattice thermal conductivities. Converged cutoff distances found for the CuGaTe_2_ chalcopyrite in our previous work^26^ (11, 6.2, and 4 Å for the second-, third-, and fourth-order force constants, respectively) were extrapolated here to the II–IV–V_2_ chalcopyrite compositions based on numbers of coordination shells (rather than absolute distances) for consistency. To check that the selected number of coordination shells remains valid for the chalcopyrite-structured II–IV–V_2_ semiconductors, we calculated the thermal conductivity for CdSiAs_2_ with one less coordination shell in the cutoff (equivalent to a cutoff distance of 5.57 Å instead of the 6.11 Å used in the reported calculation), and we obtained a difference of less than 0.09 W m^–1^ K^–1^ (∼1.2%) in the room-temperature thermal conductivity. The number of distorted structures for DFT calculations was fixed to 18 for all compositions, which is enough for convergence (see ref (26)) and well below the more than 600 DFT calculations needed, for the same cutoff distances, in the traditional approach. To simplify the workflow, our wrapper code^44^ was used in conjunction with the hiPhive program, automating the generation of distorted supercells, force calculations with VASP, and the construction of the machine-learned FCPs.

### 2.3 Boltzmann’s Transport Equation Solution

After constructing the FCP model, lattice thermal conductivities were determined by solving the BTE with the ShengBTE code.^16^ We employed the full iterative procedure to go beyond the relaxation time approximation and computed scattering times including isotopic and three-phonon scattering.

Four-phonon scattering processes were not considered in our simulations. These are very computationally expensive to include in the calculation of thermal conductivities, and we have reason to believe that their effect is relatively minor for the compounds investigated here at low to moderate temperatures. These compounds are known to have relatively high thermal conductivities and low anharmonicity, so it may be expected, in principle, that higher-order anharmonicity is generally weak. Unfortunately, this argument is not fully reliable, as it has been observed that in some solids with very high lattice thermal conductivity, the contribution from quartic anharmonicity can still be substantial.^24^ To assess the magnitude of quartic anharmonicity, we made a comparison of the forces and energies between a model with up to third-order force constants and a model including fourth-order force constants, in the case of CdSiAs_2_. This is one the compounds with the lowest lattice thermal conductivities among those reported here and therefore probably one the most anharmonic solids in the family. The contributions of fourth-order force constants to the energies and forces were computed for 300 supercells of CdSiAs_2_ whose atoms had been moved from their equilibrium positions simulating temperatures of 300 and 700 K. The suppression of the fourth-order force constants leads to RMSE values (discrepancy with respect to the full, fourth-order model) of only 7.5 × 10^–5^ eV/atom for the energies and less than 0.02 eV Å^–1^ for the forces at 300 K (the errors are only slightly larger at 700 K). This test suggests, again, that the role of quartic anharmonicity is small in these materials. Finally, the small effect of including fourth-order scattering on the lattice thermal conductivity can be expected to be partially canceled by the effect of temperature in the vibrational frequencies (which has not been included either). This cancelation effect has been reported for rocksalt and zincblende structures in ref (24). These points justify our decision not to include fourth-order effects in the simulations. But ultimately, our best argument to support the use of only up to third-order force constants is that our results lead to very good agreement with experiment, as will be shown below.

A Gaussian smearing of 0.1 eV and a dense mesh of 20 × 20 × 10 **q** points were used in all the BTE calculations, striking a balance between memory demand and the convergence of κ with the number of **q** points. In order to avoid the additional computational cost of computing Born effective charges, nonanalytical contributions (NACs) were not considered, as tests in the I–III–VI_2_ chalcopyrites showed that they had only a small effect on κ (<2.5%),^26^ and the effect can be expected to be even smaller in the more covalent II–IV–V_2_ chalcopyrites studied here.

From our calculations, the thermal conductivity is obtained as a tensor, which allows us to discuss anisotropic effects in detail by comparing the conductivity along the *c* axis, κ_*z*_ = κ_33_ with that in the *ab* plane, κ_*x*_ = κ_11_ = κ_22_. The scalar values reported represent the isotropic average, obtained as one-third of the trace of the thermal conductivity tensor, i.e., κ = (κ_11_ + κ_22_ + κ_33_)/3. Throughout this study, the calculated lattice thermal conductivity will be compared with the total experimental values (lattice and electronic) due to expected negligible electronic contributions at low–mid temperatures.

## 3 Results and Discussion

**Figure 1:**
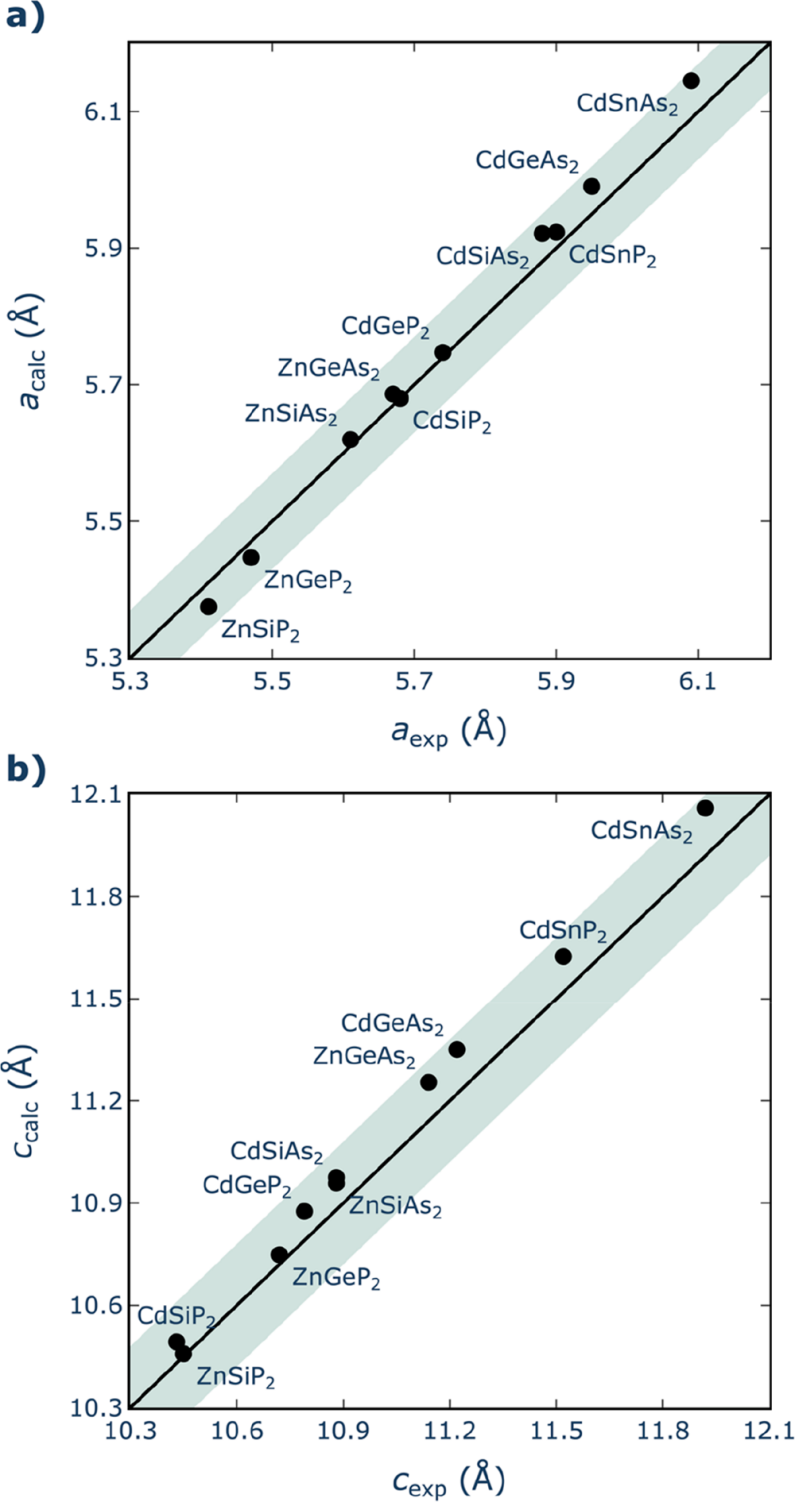
Comparison of experimental^45−53^ and DFT-calculated values of (a) *a* and (b) *c* lattice parameters. Black solid line represents perfect agreement. Green-shaded area represents deviations of ≤0.8% from experiments in either direction.

Accurate determination of cell parameters is crucial for obtaining reliable phonon properties. PBE-D3 geometry optimizations effectively reproduce the experimental lattice parameters of all compounds (Figure 1). As evidenced in our previous work with chalcopyrites,^26^ the uncorrected PBE functional overestimates the lattice parameters *a* and *c*, so the inclusion of D3 dispersion corrections allows us to obtain very low discrepancies with experimental values of ∼0.4% and ∼0.8% on average for *a* and *c*, respectively. The PBE-D3 functional provides an excellent balance between computational cost and accuracy for phonon calculations, even without addressing the main limitations of GGA in predicting electronic structures.

In contrast with the case of the I–III–VI_2_ chalcopyrites studied in ref (26), where the nature of the anion clearly had the strongest effect on the relative values of the cell parameters, for the II–IV–V_2_ chalcopyrites, the effect of the nature of the cations seems to be more significant. Interestingly, the trends are somewhat different depending on the crystallographic direction: whereas for the *a* parameter, the dominant effect is the nature of the A cation (Cd-based compounds having larger values than the Zn-based compounds), for the *c* parameter, the effect of the A cation is not so pronounced, and the nature of the B cation seems to be at least as important (with the general trend Sn > Ge > Si). As a result, there is significant structural anisotropy (as reflected in the *c*/*a* ratio), which will be discussed in more detail later, in the context of the anisotropy of the thermal conductivity (which will be characterized in terms of the ratio κ_*z*_/κ_*x*_ between the thermal conductivity in the *c* direction with respect to that in the *ab* plane).

**Figure 2:**
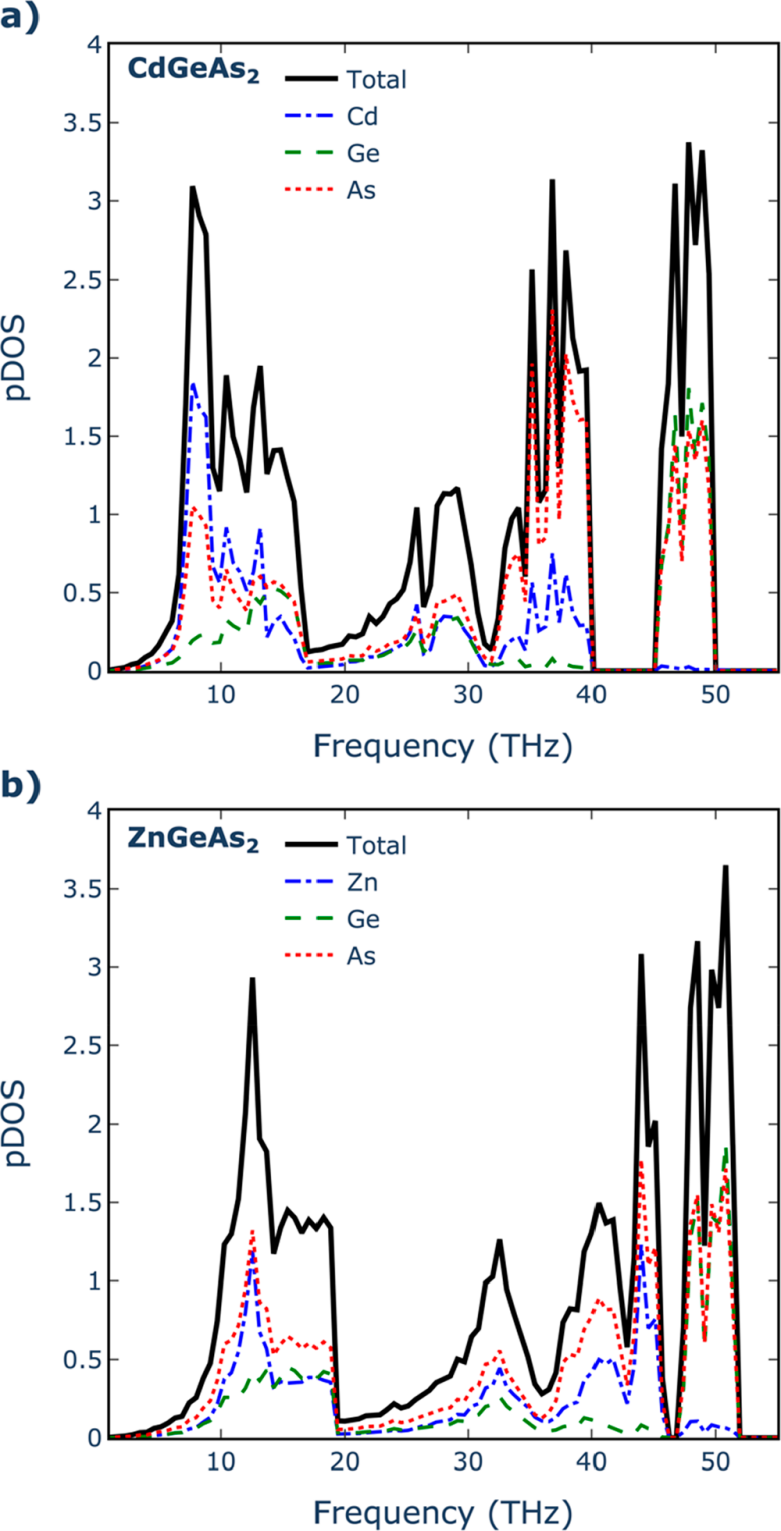
Phonon density of states (pDOS) for (a) CdGeAs_2_ and (b) CdGeAs_2_ pnictides.

**Figure 3:**
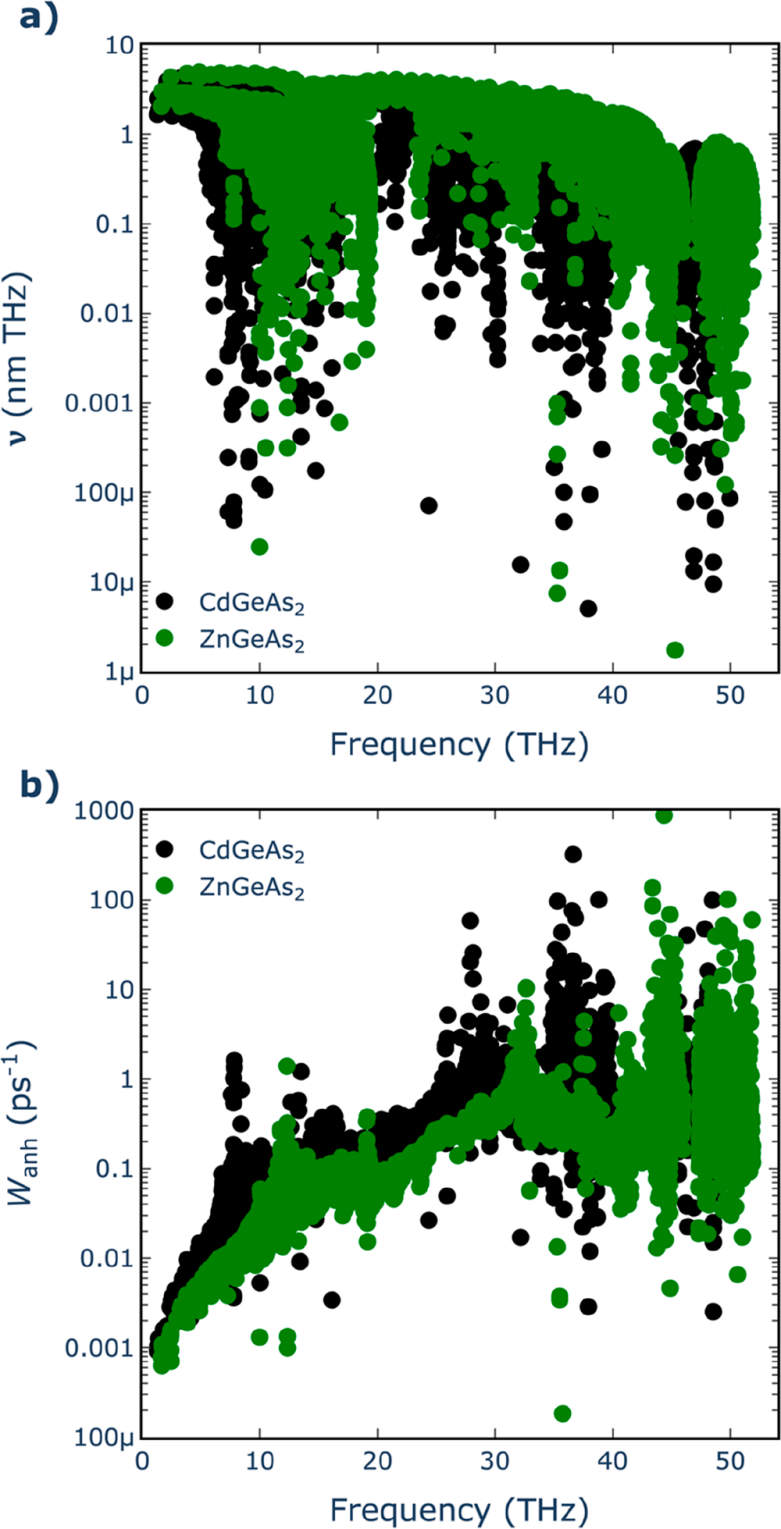
(a) Group velocities and (b) scattering rates vs mode frequency for CdGeAs_2_ and ZnGeAs_2_ pnictides.

The calculated isotropic averages of the thermal conductivities for all pnictide compositions are summarized in Table 1 at 300 and 700 K. A pattern is clearly noticed: pnictides based on Cd exhibit lower κ values compared to their counterparts based on Zn. Across the compositions involving B = Si, Ge, Sn, and X = As, P, the average value of κ for Zn-based pnictides (27.3 W m^–1^ K^–1^) is approximately twice that of Cd-based compositions (12.1 W m^–1^ K^–1^). To investigate the origin of this behavior, phonon density of states (pDOS) plots were generated in selected compounds (CdGeAs_2_ and ZnGeAs_2_) to identify the contributions stemming from the A^2+^ cations (Figure 2). Evidently, in the case of Cd, there are contributions to modes with frequencies below 1 THz, something which is not observed for the Zn-based compositions. Despite both compounds having similar elastic constants (see Supporting Information, Table S1) and thus a similar distribution of group velocities (Figure 3a), the introduction of low-frequency optical modes by Cd^2+^ cation results in high scattering rates (*W*_ahn_) at these frequencies. These scattering rates predominantly influence the thermal conductivity behavior, as illustrated in Figure 3b.

**Table 1:** Lattice Thermal Conductivities (κ) for All the Investigated Ternary Pnictide Compounds, at 300 and 700 K

	κ (W m^–1^ K^–1^)	
Compound	300 K	700 K
CdGeAs_2_	6.6	2.8
CdGeP_2_	15.9	6.7
CdSiAs_2_	7.4	3.2
CdSiP_2_	13.6	5.5
CdSnAs_2_	8.1	3.5
CdSnP_2_	21.2	9.2
ZnGeAs_2_	17.9	7.5
ZnGeP_2_	46.5	19.9
ZnSiAs_2_	20.4	8.6
ZnSiP_2_	29.7	11.8

The nature of the pnictogen X^3–^ anion also affects the thermal conductivity, with the κ values of the arsenides being generally lower than those of their phosphide counterparts. This trend can be rationalized in terms of the phonon dispersion curves (see Supporting Information, Figure S1) where the lower frequencies of the first set of optical modes in the antimonides lead to (i) a reduction of the group velocities in antimonides and (ii) a more effective overlapping between acoustic and optic modes, which means more scattering processes and higher scattering rates. On the other hand, the influence of the nature of the B cation is less predictable. For instance, B = Ge is found in both extremes of the distribution of thermal conductivities among all pnictide compositions in this study: CdGeAs_2_ (6.6 W m^–1^ K^–1^) and ZnGeP_2_ (46.5 W m^–1^ K^–1^) have the lowest and highest κ values, respectively. This does not imply that κ is unaffected by the nature of B^4+^ cations but simply that trends cannot be generalized in the same manner as observed for the ions within A and X sites, similarly to what we reported in our previous study of lattice thermal conductivities of chalcogenide chalcopyrites.^26^

**Figure 4:**
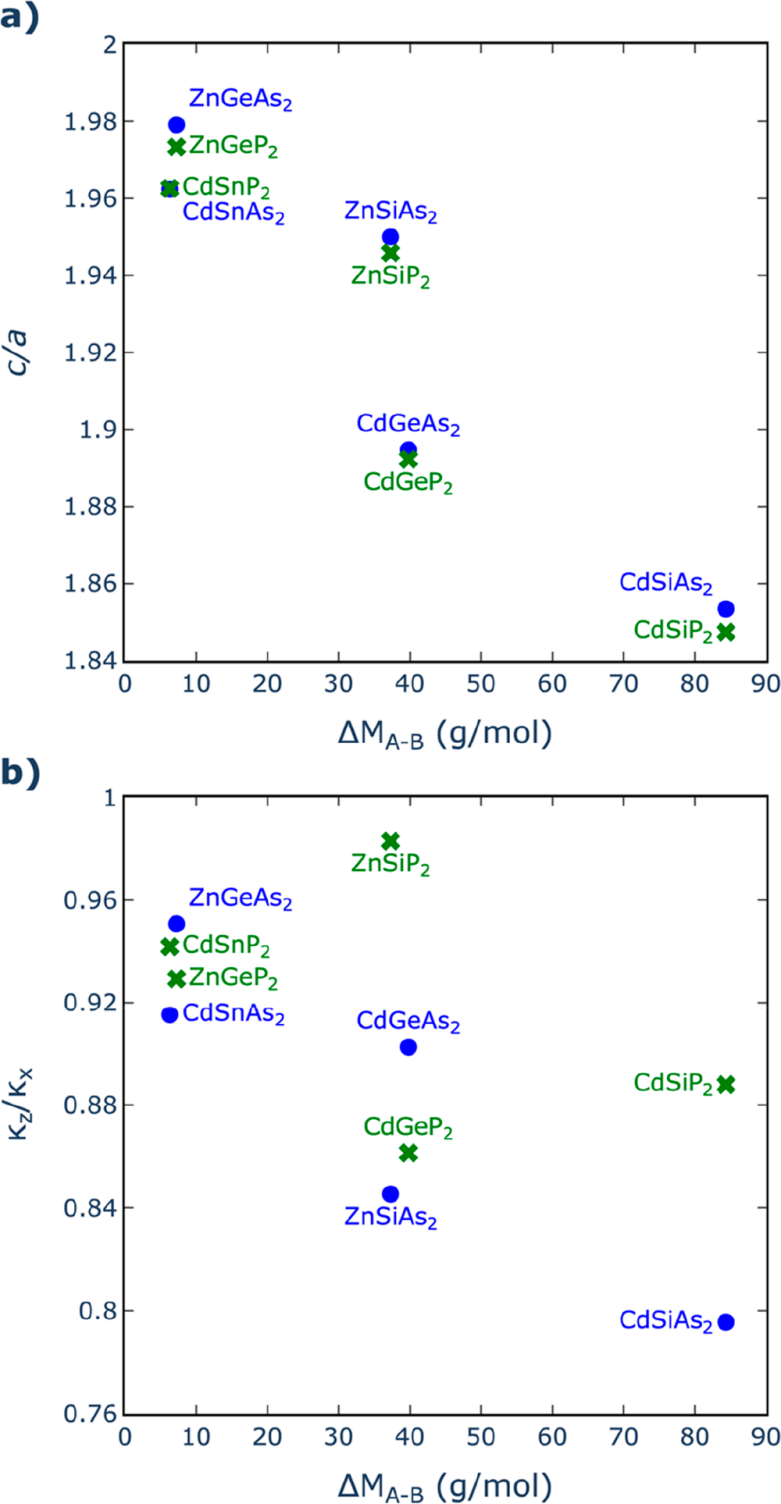
Variation of (a) the *c*/*a* ratio (characterizing the structural anisotropy) and of (b) the κ_*z*_/κ_*x*_ ratio (characterizing the thermal conductivity anisotropy) with the absolute difference in molar mass between the two cations A and B.

Having examined the trends in the isotropic average values of κ, we now focus on the anisotropic behavior. The structural anisotropy can be characterized by the deviation of the *c*/*a* ratio from the value of 2, whereas the thermal conductivity anisotropy can be characterized by the deviation of the κ_*z*_/κ_*x*_ ratio from 1. Since the origin of the anisotropic behavior in this family of materials is the distinction between the A and B cations (if the two cations were the same, we would recover the isotropic, cubic zincblende structure), it makes sense to study the variation of both *c*/*a* and κ_*z*_/κ_*x*_ versus the atomic mass difference (Δ*M*_A–B_) between the cations, as we have done in Figure 4. As a general trend, the larger the difference in mass between A and B, the more anisotropy (both structural and thermal) there is in the system. Consistently with the discussion above about the cell parameters, the structural anisotropy is not significantly affected by the nature of the anions. For example, compounds with A = Cd and B = Si exhibit the strongest deviation from *c*/*a* = 2, followed by Cd–Ge combinations. However, the thermal conductivity anisotropy is clearly affected by the nature of the anion: arsenides generally have stronger deviations from κ_*z*_/κ_*x*_ = 1 than phosphides, which can be expected due to the higher covalence of the former compared to the latter. As a result, the most anisotropic thermal conductivity is found for CdSiAs_2_, which has κ_*z*_/κ_*x*_ = 0.796 at room temperature.

**Figure 5:**
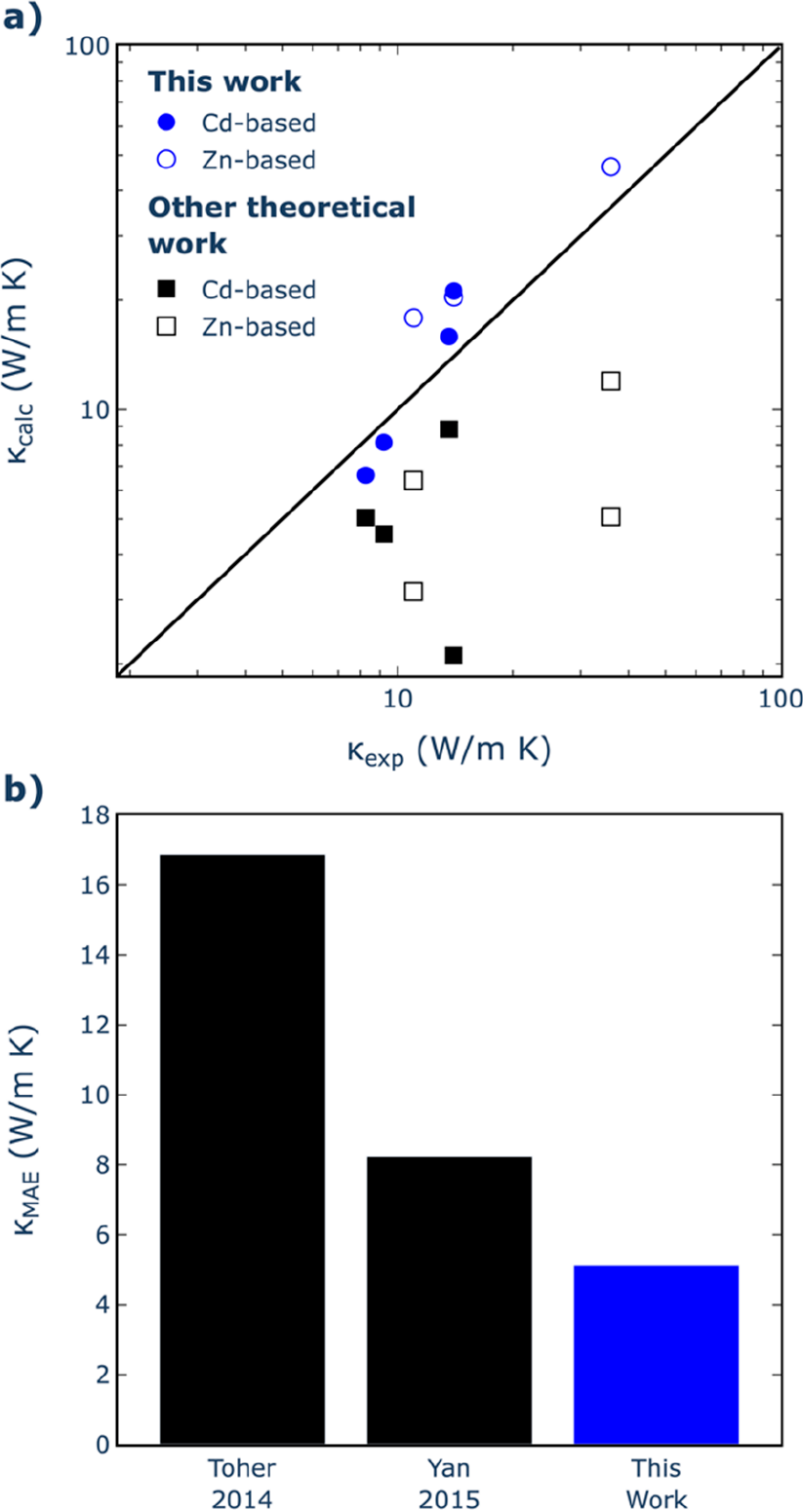
(a) Comparison of room-temperature (300 K) κ values obtained from calculations in this work (blue circles) and previous theoretical work (black squares) with available experimental data. For both cases, solid and empty symbols denote Cd- and Zn-based pnictides, respectively. Black line represents perfect agreement with experiment. (b) Mean absolute error (MAE) against experimental values in this study relative to those observed in earlier theoretical analyses. Toher 2014 is ref (54) and Yan 2015 is ref (36). Experimental data from refs (13 and 29−37).

Figure 5 presents an overview of the comparison between our findings, along with previous theoretical outcomes and experimental measurements of κ conducted at room temperature. Our calculations in this study exhibit the closest alignment with experimental data within this range of pnictide compositions (Figure 5a). This better agreement probably results from using a more advanced model for κ evaluation in comparison with prior studies. For instance, Toher et al.^54^ adopted a more approximate technique that involved combining the Slack equation^55^ with the Debye temperature and Grüneisen parameter, both extracted from DFT calculations using a quasi-harmonic Debye model. Although this technique operates independently of experimental parameters and its computational efficiency renders it suitable for a wide array of materials, it notably underestimates the thermal conductivity of all pnictide compositions. Yan et al.^36^ introduced a methodology grounded in the Debye-Callaway model,^56^ exhibiting reasonable accuracy within a single order of magnitude across a large experimental data set. While this approach led to improved results compared with those based on the Slack equation, a parameter fitting based on experimental data is required. Nevertheless, this method also tends to underestimate the κ values for all pnictides, as in Toher’s method. In contrast, the BTE-based approach used in our study enhances the calculation accuracy, boasting a mean absolute error (MAE) lower (by ∼3 W m^–1^ K^–1^) than the best previous theoretical work (see Figure 5b). This is achieved without relying on experimental input and at notably reduced computational cost when contrasted with the conventional DFT-based method for obtaining the force constants to solve the BTE.

**Figure 6:**
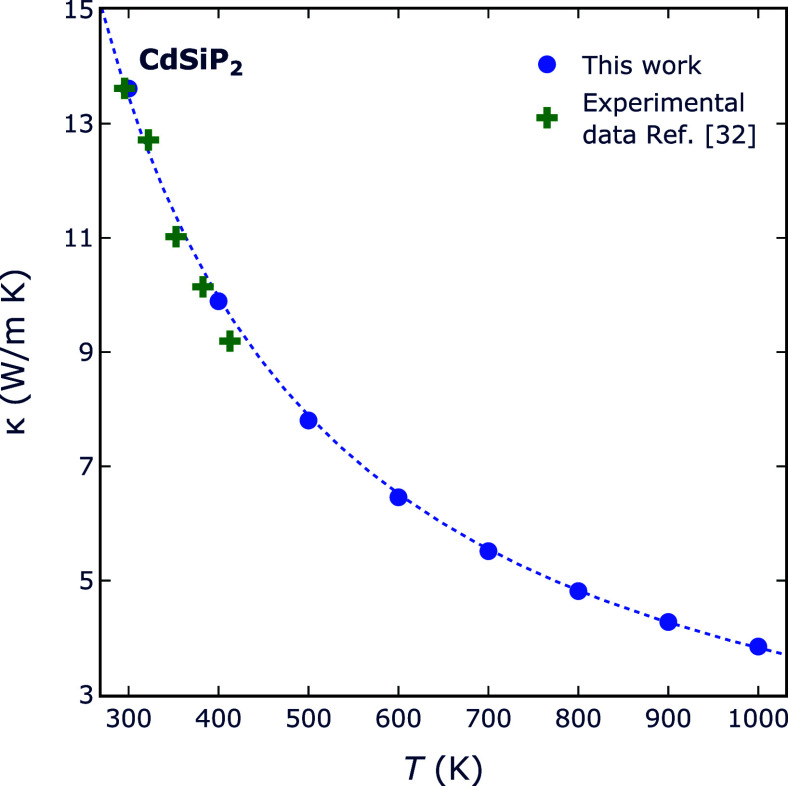
Calculated temperature variation (blue) of κ for CdSiP_2_ in comparison with the experimental data (green) reported in ref (32). The dotted line is an *T*^–1^ fit to the calculated points.

We can also contrast the projected temperature-dependent changes in κ with available experimental data from Zhang et al.^32^ on CdSiP_2_. Figure 6 depicts the characteristic variation of thermal conductivities as a function of temperature *T*. The thermal conductivity primarily stems from phonon–phonon Umklapp scattering, consequently yielding a *T*^–1^ variation. This relationship reflects the growing number of phonons that contribute to scattering as the temperature rises.

**Figure 7:**
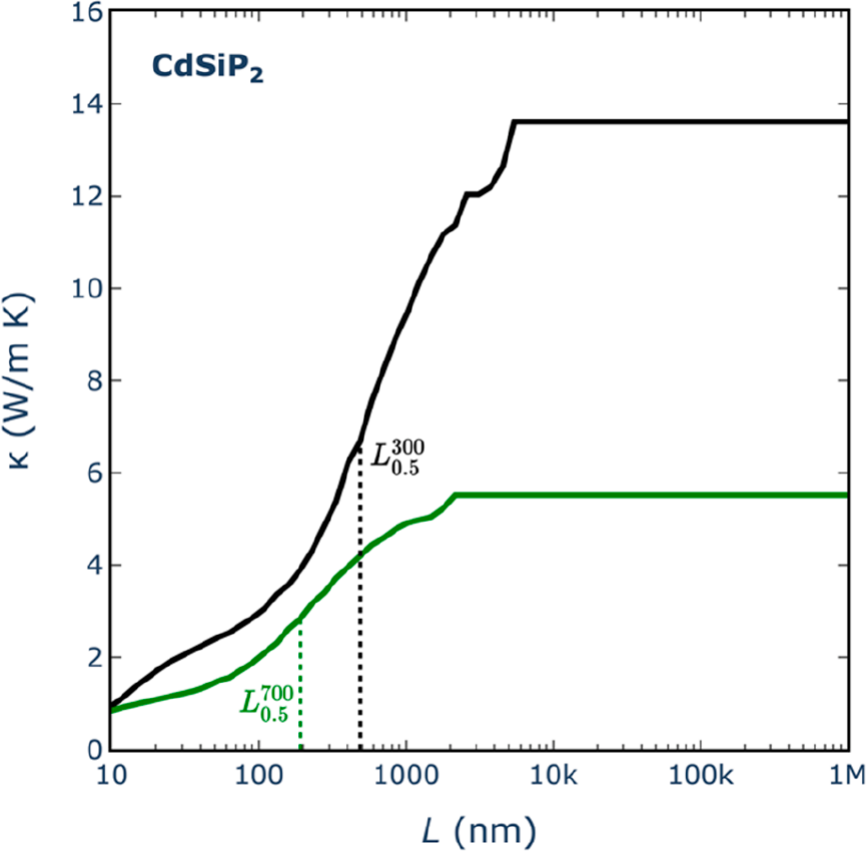
Cumulative lattice thermal conductivity from mean-free-path contributions up to distance *L* for CdSiP_2_, illustrating the effect that nanostructuring would have on thermal conductivity. Black and green lines denote *T* = 300 and 700 K, respectively.

Lastly, we explore the impact of nanostructuring on thermal conductivities using an approach that separates the contributions to κ by phonons of different mean free paths.^57^ This method, widely employed in theoretical investigations of how nanostructuring affects thermal transport in thermoelectric materials,^58−62^ estimates the κ value associated with a specific particle size *L* by considering the cumulative contributions from all mean free paths up to *L* (i.e., subtracting contributions from mean free paths exceeding the particle size). Figure 7 shows an example, for CdSiP_2_, of how the thermal conductivity is expected to vary with the particle size. It clearly illustrates that nanostructuring at the micrometer scale already exerts a significant influence on the thermal conductivity of this compound.

**Figure 8:**
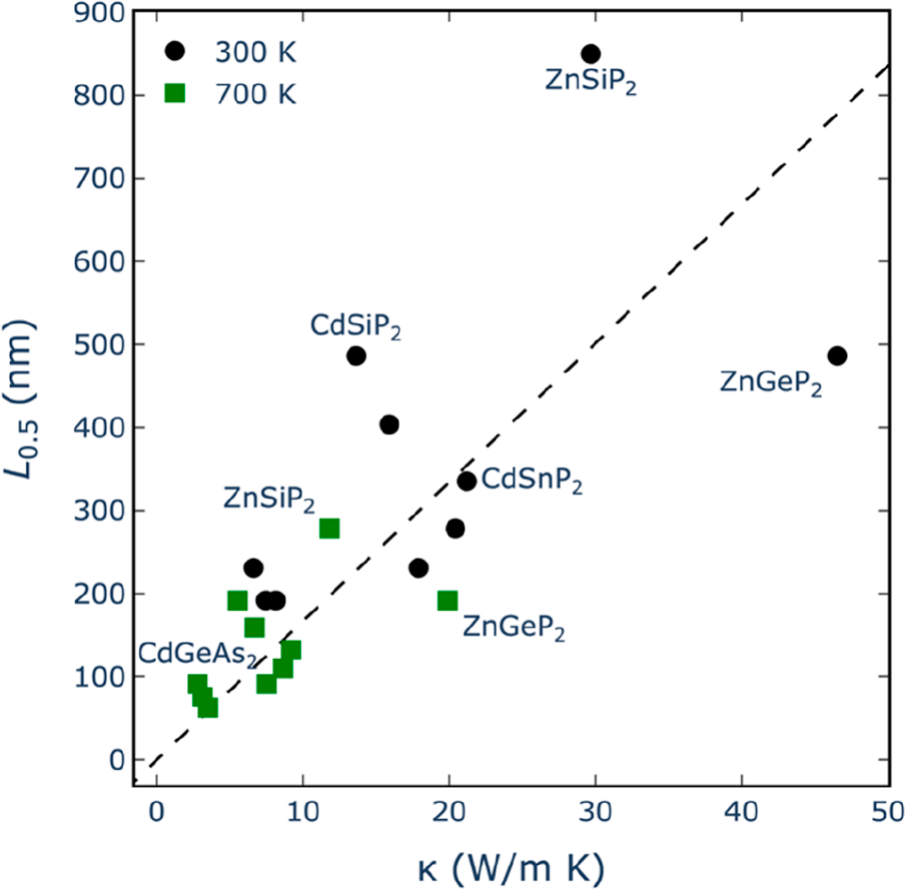
Correlation between *L*_0.5_ and (bulk) κ, including points at 300 (black circles) and 700 K (green diamonds). The dashed line corresponds to a proportionality constant of 20 nm per W m^–1^ K^–1^ between *L*_0.5_ and κ.

To quantitatively assess the trend of κ reduction due to nanostructuring across all compositions, we calculated the particle size (*L*_0.5_) that results in a 50% reduction from the bulk value, which is a function of temperature. Figure 8 shows the results at 300 and 700 K: the trend is that the lower the bulk value of κ, the smaller the *L*_0.5_. As a rule of thumb, the particle size required to reduce the thermal conductivity by half is around 20 nm per W m^–1^ K^–1^ of the bulk value of κ. This matches the trend observed for I–III–VI_2_ chalcopyrites in ref (26), although here the correlation between *L*_0.5_ and the bulk κ is not as strong as the one reported there.

The fact that the correlation between *L*_0.5_ and bulk κ is not perfect suggests a strategy to identify pnictide compounds that are particularly susceptible to reduction of thermal conductivity by nanostructuring. Compounds for which the points in Figure 8 lie above the linear regression line require less particle size reduction than the average to achieve a lower κ. For example, the room-temperature thermal conductivity of CdSiP_2_ can be halved with respect to its bulk value with particle sizes just around 500 nm. However, just halving the value of κ is not useful for thermoelectric applications of this compound because its thermal conductivity at room temperature remains too high (according to Figure 7, we would need to go to particle sizes of around 10 nm to achieve a κ of ∼1 W m^–1^ K^–1^ for this compound). For thermoelectric applications, it is more interesting to look at compounds for which the bulk κ is already low to start with, while at the same being more susceptible than average to κ reduction via nanostructuring.

At 700 K, CdGeAs_2_ is a case for which a particle size reduction to around the affordable value of 100 nm, halves the thermal conductivity, to ∼1.4 W m^–1^ K^–1^. Interestingly, a recent theoretical study^12^ has suggested that this material exhibits suitable electron transport properties for thermoelectric applications; for example, a high Seebeck coefficient above 500 μV K^–1^ at 600 K when p-doped at a concentration of 10^18^ cm^–3^, as well as moderate electrical conductivity. Using the constant relaxation time approximation with τ = 10^–14^ s, these authors estimated thermoelectric figures of merit *zT* of 0.26 at 600 K for the p-type compound at that level of doping, assuming a thermal conductivity of 4 W m^–1^ K^–1^. Our results here show that much lower thermal conductivity than that, and therefore a proportionally higher *zT*, can be achieved for this compound via nanostructuring to an affordable particle size of ∼100 nm.

## 4 Conclusions

We have performed a systematic investigation of the lattice thermal conductivities of ternary pnictide chalcopyrites using a combination of Boltzmann transport theory, density functional theory simulations, and machine-learning regression algorithms to calculate force constants. We have gained significant insights into the factors governing the thermal conductivity behavior of these II–IV–V_2_ semiconductors upon variations in chemical composition, temperature, and nanostructure.

Our study has demonstrated a clear contrast in thermal conductivity between Cd- and Zn-based pnictides. This divergence predominantly stems from the lower frequencies associated with vibrational modes involving Cd atoms. These vibrational modes exhibit an overlap with acoustic modes, leading to an amplified scattering process and reduced scattering times. While clear trends are elusive when substituting the B cation, the behavior of the pnictogen X atom follows a nonmonotonic pattern down the group. Particularly, arsenides exhibit lower thermal conductivities than their corresponding phosphides.

An evident but moderate κ anisotropy is observed, with Cd-based pnictides showing greater anisotropy than their Zn-based counterparts. This alignment echoes the structural anisotropy indicated by the *c*/*a* ratio. Accurate predictions of room-temperature lattice thermal conductivities and the correspondence between κ and temperature trends with experimental data corroborate the reliability of our findings.

Lastly, we have delved into the influence of grain size on κ by examining the particle sizes required to halve the thermal conductivity from the bulk values. We have identified interesting cases, like CdGeAs_2_, for which moderate particle size reduction can lead to thermal conductivities sufficiently low for thermoelectric applications. This is important because several II–IV–V_2_ compounds have been shown to exhibit suitable electron transport properties for thermoelectric applications but generally suffer from thermal conductivities that are too high thermal conductivities. We hope that our work will motivate further investigation of the thermoelectric potential of this family of compounds.

## Supporting Information Available

Dispersion curves, elastic properties, and phonon group velocities (PDF)
