# Supplementary material for: Theoretical Investigation of the Lattice Thermal Conductivities of II–IV–V2 Pnictide Semiconductors
Source: ACS Appl Electron Mater. 2023 Nov 22;6(5):2951–9. doi: 10.1021/acsaelm.3c01242 (PMC11137812; doi:10.1021/acsaelm.3c01242)
Supplement: Supplementary file 1 — el3c01242_si_001.pdf [file el3c01242_si_001.pdf]

Supporting Information

Theoretical investigation of the lattice thermal conductivities of II-IV-V_2 pnictide semiconductors

Victor Posligua,[a] Jose J. Plata,[a] Antonio M. Márquez,[a] Javier Fdez. Sanz,[a] Ricardo Grau-Crespo[b*]

[a]*Departamento de Química Física, Facultad de Química, Universidad de Sevilla, Seville 41012, Spain.*

[b]*Department of Chemistry, University of Reading, Whiteknights, Reading RG6 6DX, UK. Email: r.grau-crespo@reading.ac.uk*

Dispersion curves, elastic properties, and phonon group velocities

The dispersion curves (variations of the vibrational frequencies along high-symmetry directions in the Brillouin zone) are shown in **Figure S1**.

Table S1 shows the elastic constants and bulk moduli of the pnictide compounds included in our study. The elasticity of materials is connected to the behaviour of their lattice thermal conductivities. In a classical phonon gas model with isotropic behaviour, the thermal conductivity can be expressed, ignoring the frequency dependence, as: $\kappa = \frac{1}{3}C v_S l$, where C is the heat capacity, v_S is the speed of sound or group velocity of the phonons, and l is the phonon mean free path. The phonon group velocities, which depend on the softness/hardness of the material, are therefore a critical factor determining the thermal conductivity, although the presence of other factors mean that a simple linear correlation does not necessarily exist between κ and the elastic moduli within a given family of materials [1].

In Figure S2 we illustrate the clear linear relationship between the maximum group velocities and the bulk moduli, $B = [(c_{11} + c_{22} + c_{33}) + 2^* (c_{12} + c_{23} + c_{13})]/9$, across the pnictide compounds investigated in this study.

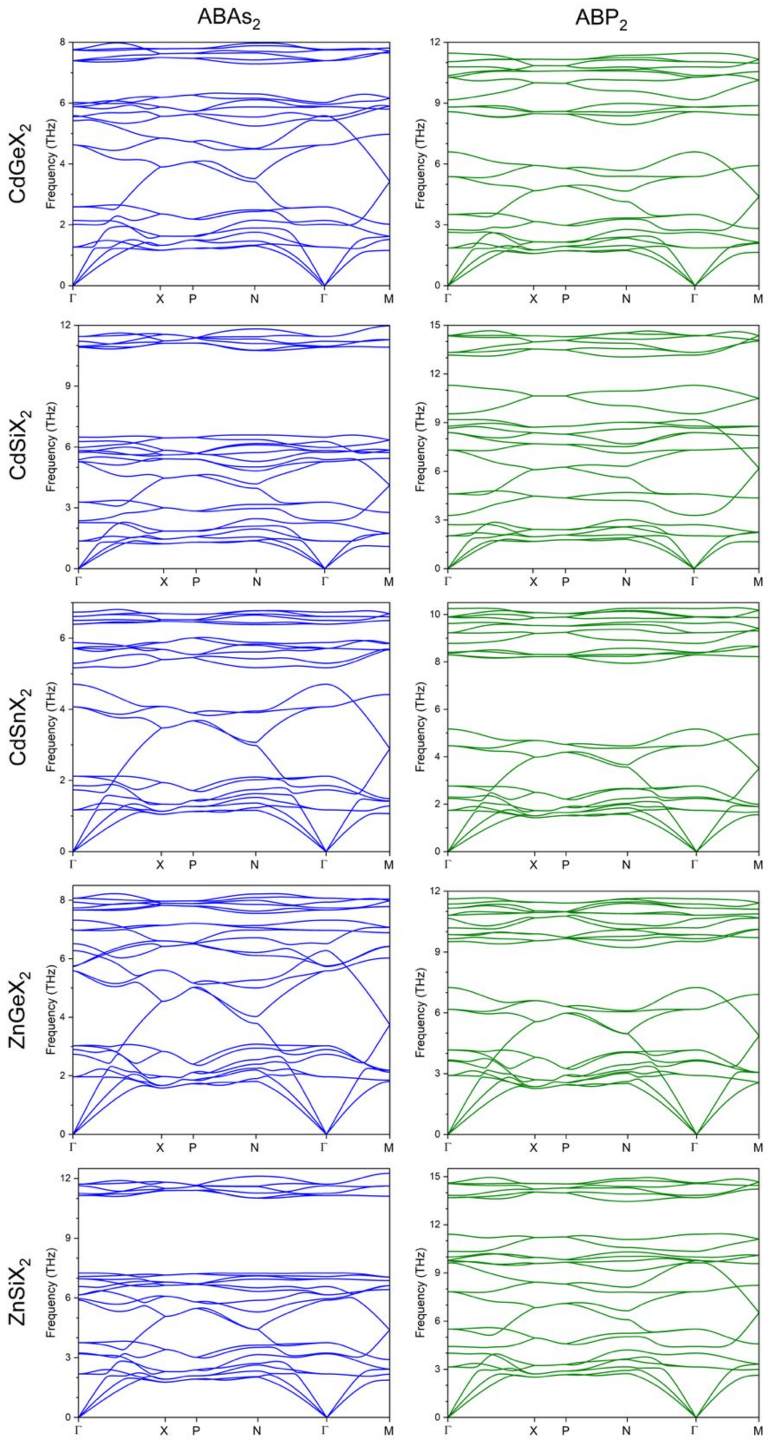

Figure S1. Phonon dispersion curves for the II-IV-V$_2$ pnictide semiconductors. Blue curves on the left are for the arsenides, green curves on the right are for the phosphides.

Table S1. Calculated elastic constants (c_{ij}) and bulk modulus (B) in GPa for each of the II-IV-V_2 pnictide semiconductors.

Materials	c_{11}	c_{33}	c_{12}	c_{13}	c_{44}	c_{55}	B
$CdGeAs_2$	85.8	76.1	44.9	47.7	34.9	35.1	58.7
$CdGeP_2$	108.0	96.1	53.3	57.6	47.4	50.4	72.1
$CdSiAs_2$	99.4	83.6	47.7	53.8	39.2	44.7	65.9
$CdSiP_2$	124.0	106.3	58.3	66.6	51.6	56.6	81.9
$CdSnAs_2$	73.9	72.3	43.9	43.9	31.5	27.8	53.7
$CdSnP_2$	90.8	88.6	51.7	52.3	40.6	41.3	64.8
$ZnGeAs_2$	99.6	100.3	46.9	48.5	49.0	51.0	65.3
$ZnGeP_2$	128.9	127.5	58.3	59.3	66.3	66.8	82.1
$ZnSiAs_2$	114.5	110.4	51.6	54.7	55.8	58.2	73.5
$ZnSiP_2$	144.5	141.1	63.4	68.2	71.8	73.8	92.2

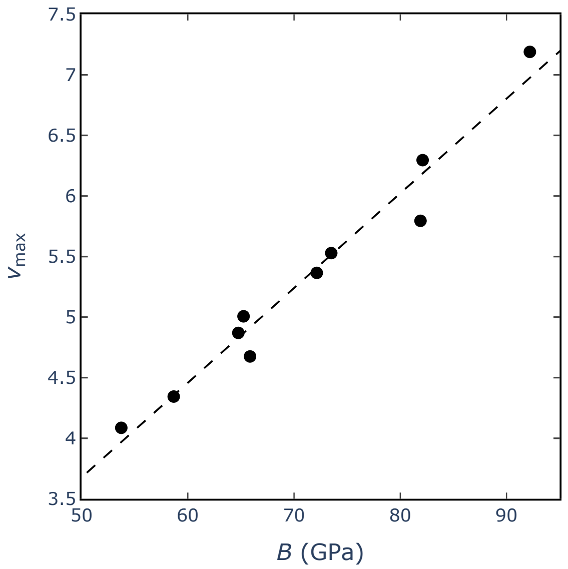

Figure S2. Maximum group velocities (v_{max}) vs bulk modulus (B) across the II-IV-V_2 pnictide semiconductors.

Reference

1. Toher, C., et al., *Combining the AFLOW GIBBS and elastic libraries to efficiently and robustly screen thermomechanical properties of solids.* Physical Review Materials, 2017. **1**(1): p. 015401.
